# Effects of inhaled corticosteroids on sputum cell counts in stable chronic obstructive pulmonary disease: a systematic review and a meta-analysis

**DOI:** 10.1186/1471-2466-5-3

**Published:** 2005-02-11

**Authors:** Wen Qi Gan, SF Paul Man, Don D Sin

**Affiliations:** 1 From the James Hogg iCAPTURE Center for Cardiovascular and Pulmonary Research, St. Paul's Hospital and the Department of Medicine (Pulmonary Division), University of British Columbia, Vancouver, B.C., Canada

## Abstract

**Background:**

Whether inhaled corticosteroids suppress airway inflammation in chronic obstructive pulmonary disease (COPD) remains controversial. We sought to determine the effects of inhaled corticosteroids on sputum indices of inflammation in stable COPD.

**Methods:**

We searched MEDLINE, EMBASE, CINAHL, and the Cochrane Databases for randomized, controlled clinical trials that used induced sputum to evaluate the effect of inhaled corticosteroids in stable COPD. For each chosen study, we calculated the mean differences in the concentrations of sputum cells before and after treatment in both intervention and control groups. These values were then converted into standardized mean differences to accommodate the differences in patient selection, clinical treatment, and biochemical procedures that were employed across original studies. If significant heterogeneity was present (p < 0.10), then a random effects model was used to pool the original data. In the absence of significant heterogeneity, a fixed effects model was used.

**Results:**

We identified six original studies that met the inclusion criteria (N = 162 participants). In studies with higher cumulative dose (≥ 60 mg) or longer duration of therapy (≥ 6 weeks), inhaled corticosteroids were uniformly effective in reducing the total cell, neutrophil, and lymphocyte counts. In contrast, studies with lower cumulative dose (< 60 mg) or shorter duration of therapy (< 6 weeks) did not demonstrate a favorable effect of inhaled corticosteroids on these sputum indices.

**Conclusions:**

Our study suggests that prolonged therapy with inhaled corticosteroids is effective in reducing airway inflammation in stable COPD.

## Background

Chronic obstructive pulmonary disease (COPD) is characterized by prominent airway inflammation [1,2]. The intensity of the inflammation strongly correlates with disease severity [3,4] and increases even further during exacerbations [5]. Moreover, increased expression of inflammatory markers in the sputum is associated with increased risk of exacerbations [6]. The attenuation of the inflammatory process, on the other hand, is associated with improvements in lung function and airway hyperresponsiveness in COPD [7]. It is possible therefore that the inflammatory process is an integral component in COPD pathogenesis and may represent an important therapeutic target in improving the health status and outcomes of COPD patients [1,2].

One potential therapy for down-regulating the inflammatory process in the airways is through the use of corticosteroids, which are potent but non-specific anti-inflammatory agents. Some in vitro studies have demonstrated that inhaled corticosteroids can modulate certain aspects of the inflammatory cascade in COPD [8,9]; however, other studies have shown less favorable results [10,11]. Despite this uncertainty, large clinical trials have shown that these medications reduce clinically relevant exacerbations by ~30% and improve health status of patients with moderate to severe disease [12]; their withdrawal, on the other hand, leads to increased risk of exacerbations and worsening of health status [13]. Since airway inflammation is associated with exacerbations [6] and since inhaled corticosteroids reduce exacerbations [12], they may also have salutary effect on airway inflammation in COPD. However, to date, the clinical studies, which have addressed this issue, have been small in size and scope and may not have had sufficient statistical power (on their own) to detect subtle but important effect of these medications on inflammatory indices in the airways. Additionally, there may be important methodologic differences between the positive and negative studies that could potentially explain the discrepancy. We, therefore, conducted a systematic review and a meta-analysis to determine whether inhaled corticosteroids do or do not suppress airway inflammation in patients with stable COPD and to explore the potential causes for the heterogeneity in reports.

## Methods

### Search for relevant studies

MEDLINE (1966–2004), EMBASE (1980–2004), CINAHL (1982–2004), and the Cochrane Databases were searched for randomized, controlled clinical trials that used induced sputum to evaluate the effect of inhaled steroids on airway inflammation in stable COPD. The search was restricted on articles published in the English language, using human participants. Subject headings included disease-specific search terms (COPD, lung diseases, pulmonary diseases, airway obstruction, obstructive pulmonary disease, chronic obstructive pulmonary disease, bronchitis, emphysema, pulmonary emphysema, or mediastinal emphysema), drug-specific search terms (glucocorticosteroids, corticosteroids, beclomethasone, budesonide, fluticasone, or triamcinolone), and laboratory method-specific search terms (biopsy, bronchoalveolar lavage, or sputum). We also scanned the bibliographies and reference lists of retrieved articles to supplement the electronic searches. We contacted the primary authors for additional data and/or clarification of data.

### Study selection and data abstraction

The primary objective of this meta-analysis was to compare the changes in sputum inflammatory indices among stable COPD patients before and after treatment with inhaled corticosteroids, using the control group in each individual studies as the referent. We chose sputum as the primary source of the analysis because there was a marked scarcity of quality studies which had evaluated the effect of inhaled corticosteroids from bronchoalveolar lavage fluid or tissue biopsy specimens. The inflammatory indices included total cell, neutrophil, macrophage, eosinophil, lymphocyte, and epithelial cell counts and interleukin (IL)-8 levels. Since the actions of oral corticosteroids may differ from those of inhaled corticosteroids, we excluded studies that evaluated the effects of oral corticosteroids on sputum inflammatory indices. From each selected article, two investigators (WQG, DDS) abstracted the following baseline information: the source of data, study design, inclusion and exclusion criteria, concomitant drugs, demographics of study participants including sample size, age, sex, current smoking status, pack-years of smoking history, predicted forced expiratory volume in one second (FEV_1_), the ratio of FEV_1 _to forced vital capacity (FVC), percent predicted reversibility with inhaled bronchodilator, the specific brand of inhaled corticosteroids and the dose as well as the duration of therapy. Cumulative dose of inhaled corticosteroids was calculated by multiplying the average daily dose by the total days of treatment. All formulations were converted to beclomethasone equivalent based on the recommendations from the Canadian Asthma Consensus Report [14]. Any questions or discrepancies were resolved through iteration and consensus.

### Statistical methods

To accommodate any differences in patient selection, clinical treatment, and biochemical procedures that were employed across the original studies, we converted the absolute mean differences in the concentrations of the inflammatory cells between the intervention and control groups into standardized mean differences. For each study, standardized mean difference was derived by dividing the mean change in the inflammatory cell concentration at follow-up visit from the baseline visit between intervention and control groups by a pooled standard deviation of the mean change [15,16]. A negative standardized mean difference indicated that the participants assigned to inhaled corticosteroids had lower cell counts compared with placebo at the end of the study phase; whereas a positive number denoted increased cell count relative to the control group. For each inflammatory cell, we tested the heterogeneity of results across the studies, using a Cochran Q test. If significant heterogeneity was present (p < 0.10), then a random effects model was used. In the absence of significant heterogeneity, a fixed effects model was used [16]. We also evaluated the potential modifying effect of cumulative dose and the duration of therapy of the trials. We reasoned that trials that had higher cumulative dose (or longer duration of therapy) defined as greater or equal to the median cumulative dose (or duration of therapy) of all the trials included in this meta-analysis would be more "positive" than those that used lower doses (or were shorter in duration). All analyses were conducted using Review Manager version 4.2 (Revman; The Cochrane Collaboration, Oxford, England) and were two-tailed in nature.

## Results

**Figure 1:**
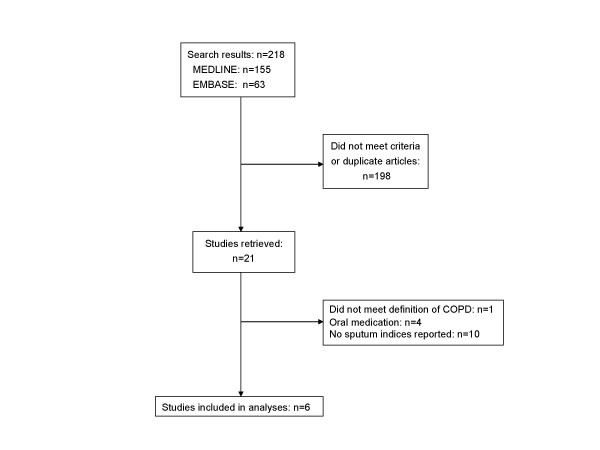
Study selection process

A summary of the search strategy is shown in Figure 1. The original search yielded 155 and 63 citations in MEDLINE and EMBASE, respectively. CINAHL and the Cochrane Databases did not contribute to the search results. The abstracts of these articles were selected and reviewed. Of these, 21 articles were retrieved for a detailed review. We excluded the study from Loppow and colleagues [17] because it included 6 patients with a positive skin prick test against at least one common airborne allergen and 4 patients who had FEV_1_/FVC > 0.7. We excluded additional 14 articles because of other reasons (Figure 1). This process left 6 original studies meeting the inclusion and exclusion criteria, which were used for the analyses [7,18-22]. The baseline information concerning the study designs is summarized in Table 1. The relevant demographic data are summarized in Table 2. All 162 patients were current or ex-smokers with post-bronchodilator FEV_1 _<70 % predicted, FEV_1 _to FVC ratio <0.7, and reversibility with bronchodilator of <15%. The medications used included budesonide, beclomethasone dipropionate, and fluticasone propionate. The study period of these trials ranged from 2 to 12 weeks.

**Table 1:** Baseline information on original studies included in the meta-analysis.

**Source**	**Setting**	**Design**	**Inclusion Criteria**	**Exclusion Criteria**	**Concomitant drugs**	**Withdrawal**	**Sputum specimen**
**Sugiura et al 2003 [7]**	NR	Randomized, placebo-controlled parallel design.	FEV_1_/FVC < 0.7; all patients wereex-smokers who had stopped smoking for at least 1 year beforethe study.	A history of perennial allergic rhinitis; positiveallergen skin prick tests and RAST assay; a history of periodicwheezing; an improvement in FEV_1 _of more than 12 % predicted oran absolute increase of 200 ml after inhalation of 200 μg salbutamol; had bronchial or respiratory tract infectionsin the month preceding the study; had taken systemic steroids in the 2 monthsbefore the study or inhaled steroids in the month beforethe study.	NR	None	NR
**Keatings et al 1997 [18]**	Outpatient clinics in different hospitals	Randomized, single-blind, crossover design with 3–7 day run in period. The clinical part of the study was single-blind, but all differential cell counting and assayswere carried out in a double blind fashion.	FEV_1_/FVC < 0.7; FEV_1 _< 70% predicted; reversibility with inhaled albuterol of <10% of predicted FEV_1_; smoking history of at least 10 pack-years; negative results on skin prick testing to four common aeroallergens.	Patients who had taken inhaled or oral steroids or who had suffered an exacerbation of their airway disease in the previous 6 weeks, or patients with any history of asthma or variability in symptoms were excluded.	Albuterol was allowed.	2 subjects	NR
**Culpitt et al 1999 [19]**	Outpatient clinic	Randomized, double-blind, placebo-controlled crossover design with a run-in period of 2 weeks.	FEV_1_/FVC < 0.7; postbronchodilat or FEV_1 _<85% predicted; reversibility with inhaled β_2_-agonist of <15% of predicted FEV_1_; smoking history of at least 20 pack-years.	Patients who had taken inhaled or oral steroids or who had suffered an exacerbation of their airway disease in the previous 6 weeks, or patients with any history of asthma or atopy or variability in symptoms were excluded.	Three subjects had concomitant treatment with albuterol (200 μg twice a day) and ipratropium bromide (40 μg twice a day), one subject with albuterol (200 μg as needed) alone.	12 subjects	Samples were considered adequate for analysis if there was < 50% squamous cell contamination.
**Confalonieri 1998 [20]**	Outpatient clinic	Randomised, controlled, open study. The clinical parts of the study was open, but all differential cell counting was carried out in a double blind fashion.	FEV_1_/FVC <88% of predicted in men and <89% in women; all patients were current smokers.	Patients who had taken inhaled or oral steroids or had suffered a respiratory tract infection in the previous three months were excluded.	None of the patients was taking theophyllines or long acting β_2 _agonists.	None	Samples were discarded if viability levels were 50% or less, or squamous contamination was 20% or more. An overall differential cell count on 500 nucleated non-squamous cells was performed by two examiners and results reported as mean of the two counts.
**Mirici et al 2001 [21]**	Outpatient clinic	Randomized, double-blind, placebo-controlled parallel design.	FEV_1 _< 70% predicted; no self-reported asthma; reversibility with inhaled terbutaline of <15% of predicted FEV_1_; current smokers.	Long-term treatment with oral or inhaled steroids within 6 months of study entry; A respiratory tract infection in previous 3 months; pregnancy or lactation, or presence of other serious systemic diseases.	β_2 _– agonists of all kinds, theophylline, and mucolytics were allowed.	10 subjects	Samples were discarded if viabilitylevels were 50% or less, or squamous contamination was 20% or more
**Yildiz et al 2000 [22]**	Outpatient clinic	Randomized, placebo-controlled parallel design with a run-in period of 2 weeks.	FEV_1_/FVC < 0.7; FEV_1 _< 70% predicted; reversibility with inhaled albuterol of <10% of predicted; smoking history of at least 10 pack-years.	Patients with any history of asthma or variability in symptoms, and patients who had taken inhaled or oral steroids or had suffered a respiratory tract infection or exacerbation in the previous 6 weeks were excluded.	All of the patients continued to inhale both salbutamol and ipatropium bromide. In 9 patients, sustained release theophylline was also administered.	None	NR

Abbreviations: FEV_1_, forced expiratory volume in 1 second; FVC, forced vital capacity; NR, not reported.

**Table 2:** The characteristics of COPD patients at baseline.

**Source**	**Number of Patients**	**Age (year)**	**Men (%)**	**Current Smokers (%)**	**Pack-years**	**FEV_1 _(% predicted)**	**Ratio (%)**	**Reversibility (% predicted)**	**Drug**	**Dose (mg/day)**	**Duration (weeks)**	**Cumulative dose (mg) ^#^**
Sugiura [7]	18^‡^	70(7)	89	0*	NR	1.2(0.4)^†^	<70	<12	Beclomethasone	0.8	4	22.4
Keatings [18]	26	45–78	60	46	>10	35.1(4.7)	<70	<10	Budesonide	1.6	2	28.0
Culpitt [19]	26	43–73	62	69	>20	49.5(16.6)	<70	<15	Fluticasone	1.0	4	56.0
Confalonieri [20]	34	58 (5)	59	100	NR	59.7(37.1)	67 (5)	NR	Beclomethasone	1.5	8	84.0
Mirici [21]	40	53(10)	75	100	26.5 (16.1)	62.0(7.4)	NR	<15	Budesonide	0.8	12	84.0
Yildiz [22]	18	64(7)	78	89	52.0 (23.4)	44.5(2.7)	57 (3)	<10	Fluticasone	1.5	8	168.0

† FEV_1_, liter;

‡ 6 patients in control group

* All subjects were ex-smokers and stopped smoking for at least 1 year.

# Cumulative dose = daily dose × days × adjusted factor for beclomethasone equivalence [14].

Continuous variables are presented as mean (SD)

Abbreviations: FEV_1_, forced expiratory volume in 1 second; FVC, forced vital capacity; ratio, the ratio of FEV_1 _to FVC; NR, not reported/not calculable.

**Figure 2:**
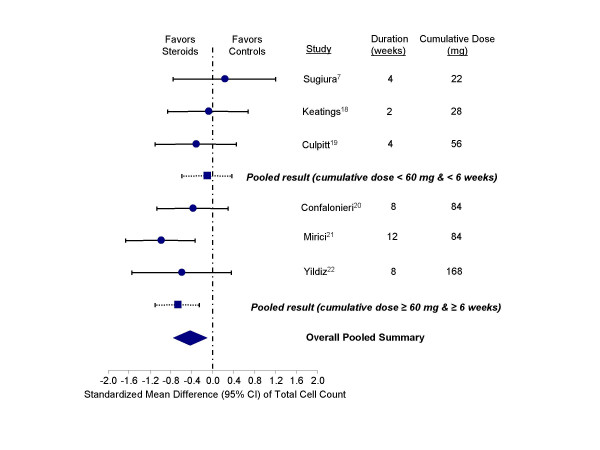
Effect of inhaled corticosteroids on total cell counts in the sputum of stable COPD patients

After treatment with inhaled corticosteroids, the total cell counts decreased. Overall, the standardized mean difference between steroid and control groups was -0.43 units (95% confidence interval, CI, -0.75 to -0.11), indicating that inhaled corticosteroids had a favorable effect in reducing total count compared with controls (test for heterogeneity, p = 0.35) (Figure 2). Importantly, the total cumulative dose of inhaled corticosteroids calculated on the basis of mean daily dose and duration of therapy made a material difference to the results. In the studies in which patients were exposed to 60 mg or greater of beclomethasone or its equivalent for the duration of the trial, inhaled corticosteroids were effective in reducing the total sputum cell count (-0.68 units; 95% CI, -1.11 to -0.26). In contrast, trials with cumulative dose of < 60 mg did not demonstrate a favorable effect of inhaled corticosteroids on this sputum index (-0.11 units; 95% CI, -0.58 to 0.37). All of the trials with the higher cumulative dose had exposed the trial participants to inhaled corticosteroids for at least 6 weeks; whereas, the trials with the lower cumulative dose was uniformly less than 6 weeks in duration.

**Figure 3:**
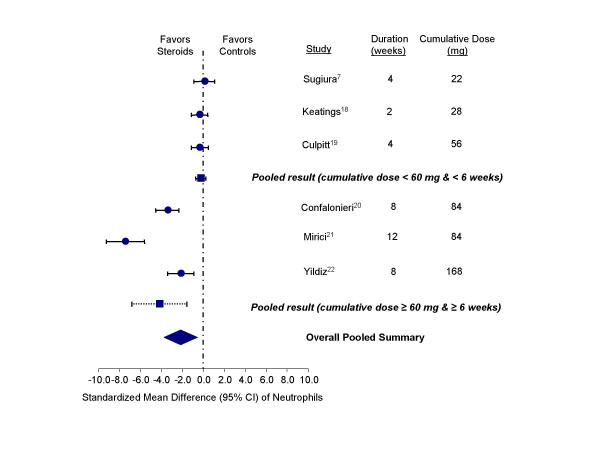
Effect of inhaled corticosteroids on neutrophils in the sputum of stable COPD patients

Inhaled corticosteroids had a salutary effect on neutrophil counts in the sputum. As compared with the control group, the standardized mean difference in those treated with inhaled corticosteroids was -2.16 units (95% CI, -3.81 to -0.50; test for heterogeneity, p < 0.001) (Figure 3). Similar to the findings on the total cell count, trials with a cumulative dose of ≥ 60 mg of beclomethasone (or at least 6 weeks of therapy) demonstrated a significant effect of these medications on sputum neutrophil count (-4.27 units; 95% CI, -6.87 to -1.66); whereas, trials with cumulative dose of < 60 mg (or less than 6 weeks of therapy) failed to demonstrate a beneficial effect (-0.26 units; 95% CI, -0.74 to 0.22).

**Figure 4:**
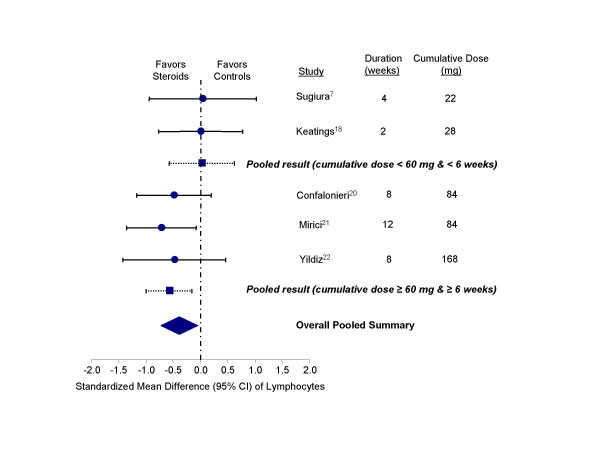
Effect of inhaled corticosteroids on lymphocytes in the sputum of stable COPD patients

Inhaled corticosteroids also reduced the lymphocyte counts in the sputum (standardized mean difference, -0.39 units, 95% CI, -0.74 to -0.05; test for heterogeneity, p = 0.58) (Figure 4). Trials with cumulative dose of ≥ 60 mg (or at least 6 weeks of therapy) demonstrated a significant effect (standardized mean difference, -0.59 units; 95% CI, -1.01 to -0.17); whereas, trials with cumulative dose < 60 mg (or less than 6 weeks of therapy) failed to demonstrate a salutary effect on this endpoint (standardized mean difference, 0.02; 95% CI, -0.59 to 0.62).

**Figure 5:**
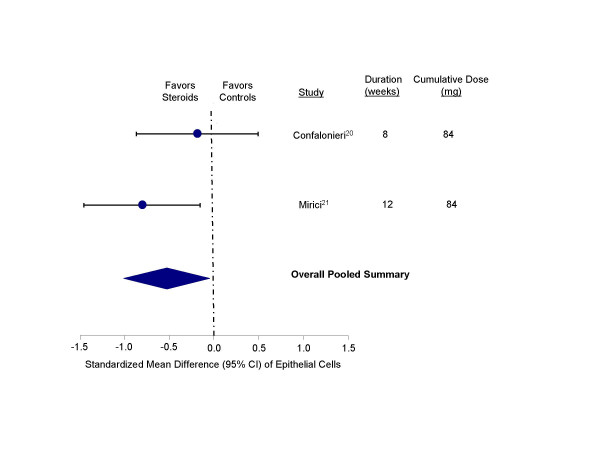
Effect of inhaled corticosteroids on epithelial cells in the sputum of stable COPD patients

**Figure 6:**
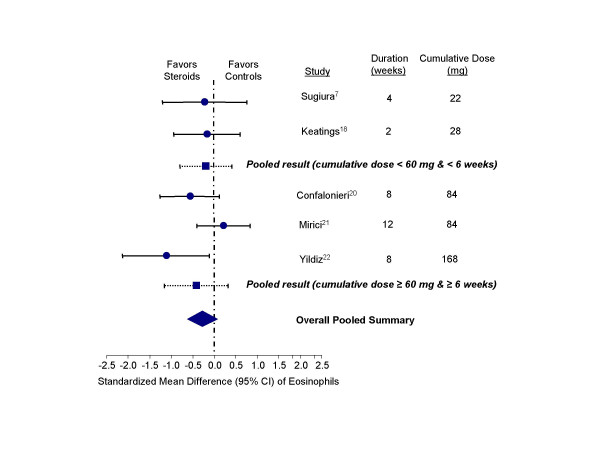
Effect of inhaled corticosteroids on eosinophils in the sputum of stable COPD patients

**Figure 7:**
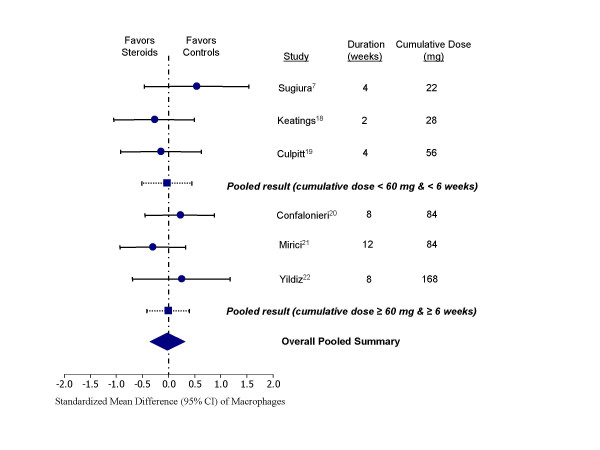
Effect of inhaled corticosteroids on macrophages in the sputum of stable COPD patients

These medications were also effective in reducing epithelial cell counts compared with the controls (standardized mean difference, -0.51 units, 95% CI, -0.98 to -0.05; test for heterogeneity, p = 0.20) (Figure 5). There was an insignificant trend towards reducing eosinophil counts in the sputum with inhaled corticosteroid therapy (standardized mean difference, -0.28 units, 95% CI, -0.62 to 0.07; test for heterogeneity, p = 0.22) (Figure 6). Inhaled corticosteroids did not appear to have any significant effect on macrophage concentrations in the sputum (standardized mean difference, -0.02 units, 95% CI, -0.34 to 0.29; test for heterogeneity, p = 0.65) (Figure 7). Inhaled corticosteroids did not have significant effect on sputum IL-8 levels (standardized mean difference, -0.22 units; 95% CI, -0.77 to 0.32; test for heterogeneity, p = 0.84).

To evaluate whether the magnitude of the reduction in the inflammatory cells was modified by the absolute levels of the inflammatory cells in the sputum at baseline, we performed a stratified analysis based on the total cell counts at baseline (see Table 3). No significant patterns were observed with any of the cell lines suggesting that baseline "cell load" in the sputum was not a predictor of response to inhaled corticosteroids.

**Table 3:** Total and differential cell counts at baseline and the standard mean difference (SMD) in cell counts between intervention group and placebo group after treatment.

**Source**	**Total cells**		**Neutrophils**		**Lymphocytes**		**Eosinophils**		**Macrophages**	
	**Number (× 10^4^/mL)**	**SMD (95% CI)**	**Number (× 10^4^/mL)**	**SMD (95% CI)**	**Number (× 10^4^/mL)**	**SMD (95% CI)**	**Number (× 10^4^/mL)**	**SMD (95% CI)**	**Number (× 10^4^/mL)**	**SMD (95% CI)**
Yildiz [22]	350.0	-0.6 (-1.6 to 0.4)	260.0	-2.2 (-3.4 to -1.0)	3.5	-0.5 (-1.4 to 0.5)	7.0	-1.1 (-2.1 to -0.1)	80.0	0.2 (-0.5 to 0.9)
Confalonieri [20]	219.0	-0.4 (-1.1 to 0.3)	158.8	-3.4 (-4.5 to -2.3)	6.6	-0.5 (-1.2 to 0.2)	6.2	-0.6 (-1.3 to 0.1)	45.0	-0.3 (-0.9 to 0.3)
Mirici [21]	196.5	-1.0 (-1.7 to -0.3)	146.5	-7.5 (-9.3 to -5.6)	1.6	-0.7 (-1.4 to -0.1)	1.6	0.2 (-0.4 to 0.8)	38.2	0.5 (-0.5 to 1.5)
Sugiura [7]	165.7	0.2 (-0.8 to 1.2)	102.9	0.1 (-0.9 to 1.1)	6.1	0.04 (-0.9 to 1.0)	4.5	-0.2 (-1.2 to 0.8)	52.0	-0.3 (-1.1 to 0.5)
Culpitt [19]	165.0	-0.3 (-1.1 to 0.5)	145.0	-0.4 (-1.2 to 0.4)	NR	NR	NR	NR	25.0	-0.2 (-0.9 to 0.6)
Keatings [18]	6.3*	-0.1 (-0.9 to 0.7)	4.3*	-0.4 (-1.1 to 0.4)	6.0*	0.0 (-0.7 to 0.8)	0.2*	-0.2 (-1.0 to 0.6)	1.8*	0.2 (-0.7 to 1.2)
Pooled Summary		-0.4 (-0.8 to -0.1)		-2.2 (-3.8 to -0.5)		-0.4 (-0.7 to -0.1)		-0.3 (-0.6 to 0.1)		-0.02 (-0.3 to 0.3)

* cell count/ml

Abbreviations: NR, not reported/not calculable.

**Figure 8:**
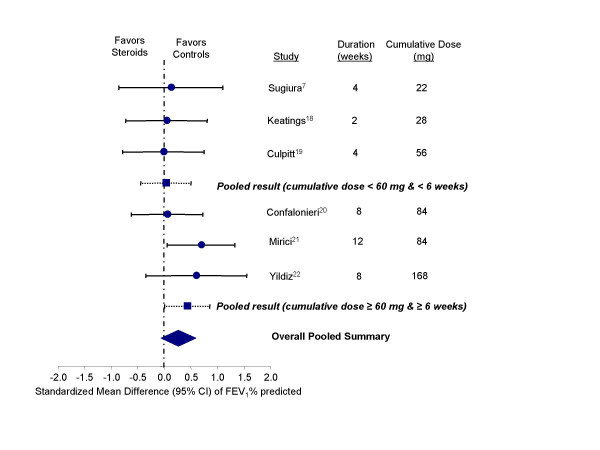
Effect of inhaled corticosteroids on FEV1_1_% predicted in stable COPD patients. Abbreviation: FEV_1_, forced expiratory volume in one second

After treatment with inhaled steroids, lung function improved slightly but neither the improvement in FEV_1 _nor FVC reached statistical significance. For predicted FEV_1_, the overall standardized mean difference was 0.26 units, 95% CI, -0.06 to 0.57 (test for heterogeneity, p = 0.62) (Figure 8). For predicted FVC, the overall standardized mean difference was 0.31 units; 95% CI, -0.09 to 0.70 (test for heterogeneity, p = 0.23).

## Discussion

By combining data across the clinical studies, we increased statistical power to demonstrate a salutary effect of moderate to high doses of inhaled corticosteroids on some inflammatory indices in the sputum of patients with stable COPD. Over a short term, these medications reduced neutrophil, lymphocyte and epithelial cell counts in the sputum of stable COPD patients. They had smaller (and insignificant) effect on sputum eosinophils and IL-8. They had little effect on sputum macrophages. Although the magnitudes of these reductions were relatively small, they may explain why inhaled corticosteroids decrease cough and sputum production [23], reduce exacerbations [24], and hospitalizations [25].

We also found that duration of therapy and total cumulative dose, which are related constructs, made a material difference to the overall results. Short trials (less than 6 weeks in duration) were uniformly "negative"; while longer term trials (at least 6 weeks of therapy) were mostly positive. Similarly, trials that exposed the patients to higher cumulative dose were more "positive" than those that exposed patients to lower dose. This suggests that duration of therapy and total cumulative doses may be important determinants of the effect of inhaled corticosteroids on airway inflammation.

Although corticosteroids delay neutrophil apoptosis and may increase neutrophil survival [11,26], they also have significant inhibitory action on neutrophil performance. Likely through the annexin-I (lipocortin-1) pathways, for instance, corticosteroids interfere with neutrophil chemotaxis, adhesion, transmigration, oxidative bursts, and phagocytosis, thereby down-regulating the overall inflammatory cascade [9,27]. Indeed, Llewellyn-Jones and co-workers [28] showed that 4 weeks of inhaled fluticasone therapy can significantly reduce sputum chemotactic activity for neutrophils and increase its elastase inhibitory capacity in patients with well-characterized COPD. These data suggest that inhaled corticosteroids can reduce recruitment and/or adhesion of neutrophils to the airways of COPD patients, thereby lowering the overall concentration of these cells in COPD airways.

Superficially, the present data on sputum eosinophils appear to be inconsistent with the known effect of corticosteroids in general on eosinophils. Many experiments have shown that eosinophils are exquisitely sensitive to corticosteroids [29,30]. The current data, however, suggest otherwise. Several studies have demonstrated that among COPD patients with irreversible airflow obstruction (as was the case for a majority of patients enrolled in the original studies contained in this meta-analysis), eosinophils are present in relatively small quantities in the sputum of such patients [10,31]. In most COPD patients, eosinophils account for less than 2% of the total cells in the sputum. This could have introduced a "floor" bias wherein the overall signal to the noise ratio for eosinophils may have been too small to detect subtle but important effect of inhaled corticosteroids on these cells. Although by combining data from these published studies we increased the power of the present analysis to detect salient changes in the inflammatory indices of the sputum, we may still have had insufficient power for analyses of cells with a relatively small signal. Our analysis may also have had insufficient power to assess the effects of inhaled corticosteroids on FEV_1_. Although there was a trend towards improvement, we did not find a statistically significant effect of inhaled corticosteroids on FEV_1_. Larger randomized trials have demonstrated, however, that inhaled corticosteroids significantly improve FEV_1 _over the first three to six months of therapy [25,32-34], suggesting that for certain endpoints our present analysis still lacked sufficient power. Therefore, the "negative" associations must be interpreted cautiously. It is also important to note that none of the studies included in the present review evaluated the effects of inhaled corticosteroids on the function or performance of inflammatory cells in the airway. Thus, we can not discount the possibility that these medications could have salutary effects on the functional performance of these cells.

In the present review, we did not include randomized studies that used bronchoalveolar lavage (BAL) or bronchial biopsies to measure inflammatory cells in the airways. However, in one study, Balbi and colleagues [35] observed significant reductions in the total number of cells, neutrophil counts, IL-8, and myeloperoxidase levels in the BAL fluid of COPD patients after 6 weeks of inhaled beclomethasone therapy. A similar finding was observed and reported by Thompson and coworkers [36]. In another experiment, Hattotuwa at al [23] randomly treated a group of COPD patients with 3 months of inhaled fluticasone propionate (1 mg/d) or placebo. The group that received fluticasone had significantly fewer mast cells in the subepithelial layer as well as a reduced ratio of CD8 to CD4 positive cells in the epithelial layer than those treated with placebo. Most importantly, the fluticasone group had significant improvements in cough and sputum scores and decreased use of reliever medications and experienced fewer exacerbations than did the placebo group [23]. Verhoeven et al [37] evaluated 23 patients with COPD and randomly treated 10 patients to fluticasone (1 mg/d) and the remainder to placebo. After 6 months, fluticasone treatment resulted in a significant reduction in the number of MBP and CD68 positive cells in the lamina propria and reduced tryptase levels in the epithelium. In addition, there was a trend towards fewer CD3, CD4 CD68 positive cells in epithelium of the group treated with fluticasone compared with the group treated with placebo [37]. The results from the BAL and bronchial biopsy studies largely support data from the sputum studies and are consistent with the notion that inhaled corticosteroids reduce airway inflammation in COPD.

We also did not include studies that used systemic corticosteroids. Barcyk and colleagues [38] have reported that oral prednisone therapy (0.5 mg/kg/d) for 2 weeks significantly reduced myeloperoxidase levels in the sputum of COPD patients. Brightling and colleagues [39] showed that 2 weeks of oral prednisone therapy resulted in fewer eosinophils in the sputum of COPD patients. Similar findings were reported by Fujimoto and colleagues [40]. These data suggest that oral prednisone can reduce certain components of airway inflammation (e.g. eosinophils) in COPD; however, most of the studies were very short in duration, which makes it difficult to compare these data against those studies that used inhaled corticosteroids.

Although in the present review, we could not adequately determine the effects of tobacco smoke exposure on the relationship between inhaled corticosteroids and airway inflammation, there is a growing body of evidence to suggest that active smoking may attenuate the effectiveness of corticosteroids in suppressing airway inflammation. Active smoking increases oxidative stress and up-regulates the production of various pro-inflammatory cytokines including Il-6, IL-8, IL-1β and monocyte chemoattractant protein-1 in airways, which may through a series of complex pathways lead to a state of steroid resistance [41]. Additionally, cigarette smoke may reduce histone deacetylase activity and its expression in alveolar macrophages, making these cells relatively resistant to corticosteroids since one of the principal targets of corticosteroid action is by switching off gene expression of inflammatory genes through the recruitment of histone deacetylases [41]. Therefore smoking cessation remains the single most important intervention in COPD management. Inhaled corticosteroids should be considered as a possible adjunctive therapy in patients who remain symptomatic despite smoking cessation.

There are certain limitations with the present analysis. First, although we used stringent entry criteria in order to minimize the heterogeneity in the research methods employed by each of the selected study, there were still some variations in the study design, the exposure medications, and the target population across the original studies. However, the differences in the characteristics of the studies were relatively small and unlikely to have materially affected the overall findings of the current review. We also contacted the primary authors to clarify any ambiguities or to obtain additional data, where necessary, to further minimize the "noise" inherent to meta-analyses. Moreover, to accommodate various differences in the methodology of data collection and laboratory techniques employed across the original studies, we converted the individual data into standardized mean estimates, which enhanced the comparability of data across the original studies. Second, it is possible that corticosteroid therapy could have affected the volume of sputum recovery, decreasing the total sputum cell counts in those patients exposed to this therapy. To mitigate this possibility, the cell counts were expressed as cells per volume of sputum recovered.

## Conclusions

In summary, the present meta-analysis suggests that inhaled corticosteroids when used for longer than 6 weeks can significantly reduce neutrophil counts and other inflammatory indices in the sputum of patients with stable COPD. Large randomized controlled trials are needed in the future to confirm these early findings and to determine whether these salutary effects persist longer than 3 to 4 months of therapy.

## Abbreviations

COPD chronic obstructive pulmonary disease

FEV_1 _forced expiratory volume in 1 second

FVC forced vital capacity

SD standard deviationIL-8 interleukin-8

## Competing interests

DDS and SFP have received honoraria for speaking engagements from GlaxoSmithKline (GSK) & AstraZeneca, and have received consultation fees and research funding from GSK. However, no part of this work was financed by these companies. This work was funded by Canada Research Chair and a Michael Smith/St. Paul's Hospital Foundation Professorship in COPD.

## Authors' contributions

All the authors contributed to the design and implementation of the study. Data analyses were performed by WQG and DDS. All authors contributed to the write-up of the manuscript.

## Pre-publication history

The pre-publication history for this paper can be accessed here:


